# Hemizona Assay and Sperm Penetration Assay in the Prediction of IVF Outcome: A Systematic Review

**DOI:** 10.1155/2013/945825

**Published:** 2013-10-21

**Authors:** Paraskevi Vogiatzi, Charalampos Chrelias, David J. Cahill, Maria Creatsa, Nikos Vrachnis, Zoe Iliodromiti, Demetrios Kassanos, Charalampos Siristatidis

**Affiliations:** ^1^Assisted Reproduction Unit, 3rd Department of Obstetrics and Gynecology, University of Athens, Attikon Hospital, Rimini 1, Chaidari, 12642 Athens, Greece; ^2^3rd Department of Obstetrics and Gynecology, University of Athens, Attikon Hospital, Rimini 1, Chaidari, 12642 Athens, Greece; ^3^School of Clinical Sciences, University of Bristol, Level D, St Michael's Hospital, Bristol BS2 8EG, UK; ^4^2nd Department of Obstetrics and Gynecology, University of Athens, Aretaieion Hospital, Vasilissis Sofias 76, 11528 Athens, Greece

## Abstract

The limited predictive value of semen analysis in achieving natural conception or in IVF outcome confirms the need for sperm function tests to determine optimal management. We reviewed HZA and SPA predictive power in IVF outcome, with statistical significance of diagnostic power of the assays. HZA was readily efficient in predicting IVF outcome, while evident inconsistency among the studies analysed framed the SPA's role in male fertility evaluation. Considerable variation was noted in the diagnostic accuracy values of SPA with wide sensitivity (52–100%), specificity (0–100%), and PPV (18–100%) and NPV (0–100%) together with fluctuation and notable differentiation in methodology and cutoff values employed by each group. HZA methodology was overall consistent with minor variation in cutoff values and oocyte source, while data analysis reported strong correlation between HZA results with IVF outcome, high sensitivity (75–100%), good specificity (57–100%), and high PPV (79–100%) and NPV (68–100%). HZA correlated well with IVF outcome and demonstrated better sensitivity/specificity and positive/negative predictive power. Males with normal or slightly abnormal semen profiles could benefit by this intervention and could be evaluated prior to referral to assisted reproduction. HZA should be used in a sequential fashion with semen analysis and potentially other bioassays in an IVF setting.

## 1. Introduction

Male fertility is considered to be affected by genetic disorders, congenital or acquired urogenital abnormalities, varicocele, infection, endocrine disturbances, immunological and/or lifestyle factors, environmental assaults, and idiopathic factors with no demonstrable aetiology [1]. Fifteen percent of all couples of reproductive age are affected by infertility [2], with male factor being solely responsible in about 20% of these and a contributory factor in another 30%–40% [3]. The latter together with the lasting debate over sperm quality and density decline [4–7] intensifies the need for accurate and rapid evaluation of sperm function in assisting clinical decisions, that is, whether natural conception is feasible or medical support should be sought for reproduction, taking the age of the woman and the duration of infertility into account. 

Semen analysis is the basis of primary male investigation, and in certain cases, such as in severe oligozoospermia, semen parameters are of absolute importance and should be strongly considered in clinical management by implementing the World Health Organization (WHO) and the European Society of Human Reproduction and Embryology (ESHRE) guidelines and recommendations [8]. However, the limited predictive value of semen analysis in achieving natural conception or in assisted reproductive technology (ART) outcome such as in in vitro fertilization (IVF) [9–12] confirms the need for sperm function tests, as well as in cases of oligoasthenoteratozoospermia or idiopathic infertility when knowledge of sperm functional capacity is a requisite for determining optimal management. 

The sperm penetration assay (SPA) was introduced by Yanagimachi et al. [13] and involved the use of surrogate ova from golden hamsters, stripped of the zona pellucida to allow interspecies interaction. SPA evaluates sperm function capacity by examining sperm competence in distinct biological processes required for fertilization; capacitation, acrosome reaction, spontaneous recognition of and fusion with vitelline membrane to the end-point of chromatin decondensation [13]. The outcome of this assay is hamster oocyte penetration rate expressed as the percentage of ova with positive penetration divided by the total number of ova, in control and test samples (supplemental Figure 1 (see supplemental Figure 1 in Supplemntary Material available online at http://dx.doi.org/10.1155/2013/945825)). Positive penetration is confirmed through the presence of swollen sperm head with a visible tail or male pronucleus, through microscopical examination of the ooplasm. Correspondingly, the concept of sperm-zona pellucida binding assay was first introduced by Liu et al. [14], who pioneered the competitive zona binding (CBZ) test, and Burkman et al. [15] who established the hemizona assay (HZA) the same year. Franken et al. [16] described salt storaging of human oocytes, while Morroll et al. [17] initiated the utilization of cryostored oocytes for this assay. HZA is a functional homologous model of gamete interaction that uses nonviable, bisected human oocytes to examine spermatozoa capacity to bind to glycoprotein receptors ZP3/ZP4 on the zona surface and undergo activation and acrosome reaction in an appropriate timed manner. The main principle of this assay is the assessment of sperm-zona binding in vitro and its numerical interpretation by the calculation of the hemizona assay index (HZI) value (supplemental Figure 2). It has been estimated that defective sperm-zona binding and zona penetration are among the most common causes of fertilization failure and that within the oligozoospermic population around 80% of males produce spermatozoa that are unable to interact normally with zona pellucida [18–21]. Both SPA and HZA have the potential to represent male fertility indicators, as both techniques reflect certain biological requisites for reproduction; a sequence of functional conditions is required for the sperm to interact and fuse with the ovum. 

The validity of any diagnostic test is based on a set of criteria according to ESHRE: sensitivity (to produce few false-negatives), specificity (to produce few false-positives), complexity, time, and cost effectiveness, and positive (PPV) and negative (NPV) predictive values (in terms of fertility and fertilization rates) [9]. The aim of the present study was to review and appraise the published evidence to date on two major techniques: SPA and HZA. We reviewed their predictive power in IVF outcome and performed an assay overview in terms of protocol standardization, to determine whether these tests could be incorporated in routine male fertility assessment and whether they could be used as a tool for predicting fertilization potential.

## 2. Materials and Methods

### 2.1. Search Strategy

 This systematic review was conducted according to the PRISMA guidelines [22] and the initial protocol agreed by all authors. Studies in English language from 1976 to August 2012 were compiled with no study design restrictions, using the following search algorithms in two major scientific/medical databases (Medline/PubMed, ScienceDirect):

- “sperm penetration assay” OR “SPA” OR “HEPT” OR “ZHEPT” OR “HEPA” OR “HOPT” OR “hamster test” AND “IVF” OR “in vitro fertilization” OR “in vitro fertilisation” OR “assisted reproduction” OR “Reproductive Techniques, Assisted” OR “ART”
- “hemizona assay” OR “HZA” OR “sperm zona test” AND “IVF” OR “in vitro fertilization” OR “in vitro fertilisation” OR “assisted reproduction” OR “Reproductive Techniques, Assisted” OR “ART”.

Search results were confirmed through the SCIRUS database. There were no MeSH terms assigned to the specific assays. Reference lists of relevant articles were hand-searched for potentially eligible studies. 

### 2.2. Screening

To minimize bias (extraction, recording, conformity, and retrieval), three authors (P. Vogiatzi, C. Siristatidis, and M. Creatsa) performed the primary evaluation of titles and abstracts identified through the publication identification process, and each author provided a list of potentially eligible studies. Those three authors extracted the data independently, using a preagreed data extraction form. Collected data included general information (title, author, year, journal, and clinical setting), study characteristics (design, inclusions/exclusions), participants' characteristics (cause and duration of preexisting infertility, semen parameters, and protocols for ovarian stimulation), assay parameters (SPA/HZA and fertilization rate cutoff values, different culture media and sperm enhancers used, incubation lengths, hemizona sources, and preservation methods), and results (number of participants, reference population, specificity and sensitivity of the assay, and PPV and NPV as reported or calculated by the data sets provided by the study group). If multiple publications using the same cohort were identified, the most recent or more complete publication was used for data extraction. Two authors (P. Vogiatzi and C. Siristatidis) performed the final selection of the potential eligible studies; conflicts were resolved by team consensus.

### 2.3. Eligibility

Studies comparing HZA and SPA results with outcome parameters in an IVF setting were considered to examine the validity of these tests. Studies had to be published in a scientific/medical journal and be easily accessed through electronic means or retrieved through a printed library version. Case series/reports, animal studies, or articles published on independent websites were excluded. Modifications in methodology that greatly deviate from the conventional protocol and interventions that could interfere with the results were also weighed up. Statistical analysis methods and presented data were also taken into account, in terms of providing sensitivity/specificity and PPV/NPV or otherwise reporting the appropriate data sets in order to calculate these values and determine the resulting diagnostic accuracy, since these are the parameters that define the validity of diagnostic tests according to ESHRE [9]. Eligible studies were excluded for the following reasons: protocols including pre-treatments or interventions that could affect the results, studies with other than fertilization rate as a primary outcome or through natural conception and other assisted conception techniques (intrauterine insemination-IUI, intracytoplasmic sperm injection-ICSI, subzonal sperm insemination-SUZI), and essential data for diagnostic accuracy not shown and grouped data for true positives/negatives and false- positives/negatives missing. Where applicable, data was analyzed as published by each study group; reevaluations were not included. Reported oocyte immaturity and laboratory/equipment failure were disregarded if these results were already incorporated in the presented data. The outcome measure was fertilization outcome through IVF compared to SPA/HZA results with statistical significance by reporting specificity, sensitivity, PPV, and NPV values for diagnostic power of the assays.

## 3. Results

**Figure 1:**
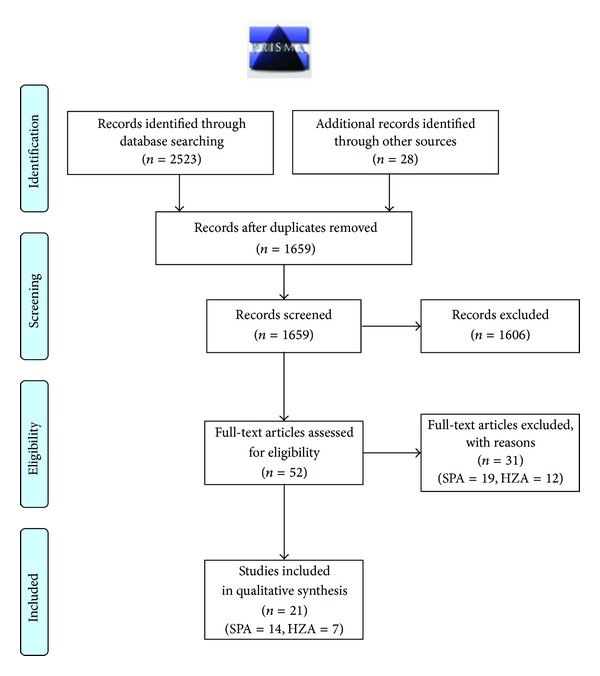
PRISMA flow diagram of study inclusion process.

The initial search yielded 2551 potentially relevant studies that were reduced to 1659 by removing duplicates (Figure 1). All titles and abstracts were screened to exclude irrelevant publications, resulting in 52 potentially eligible studies. The full-text articles were assessed, and 21 manuscripts (14 on SPA and 7 on HZA) were identified to provide data corresponding to the research question [23–43]. Characteristics of the studies included publication date, study design, and size as well as patient characteristics and experimental interventions (capacitation medium type and pre-incubation lengths/oocyte source and preservation means) which are presented in Tables 1(a) and 1(b), for SPA and HZA, respectively. Assay methodology researched in this review has been presented in supplemental Figures 1 and 2, for SPA and HZA, respectively. There were no limitations in participants' age, and fertility status was either unknown or defined through conventional semen analysis (hereby reported as normal/abnormal semen parameters), while control groups consisted of sperm samples from donors with proven fertility and normal semen parameters. A summary of assay and fertilization rate cutoff values, along with sensitivity/specificity, PPV/NPV, are presented in Tables 2 and 3, for SPA and HZA, respectively. Statistical values were either extracted from the manuscripts or calculated through the reported values to provide the diagnostic accuracy parameters for each test. 

The addressed usefulness and validity of SPA were presented with major discrepancies in protocols, statistical analysis, and outcome. The inconsistencies in capacitation medium type and incubation lengths in SPA methodology are illustrated in Table 1(a). Protocol variation across studies was evident in the range of SPA cutoff values used (Table 2), final sperm concentration for the assay ranging from 0.4 to 20 × 10^6^/mL, in contrast to the suggested 5 × 10^6^ by the WHO manuals [44] and sperm preparation methods [45, 46]. Second and third generation SPA promoted the addition of mild enhancers, hyperosmotic medium, albumin, follicular fluid, TEST yolk buffer, calcium ionophore, progesterone, and pentoxyfline, at variable concentrations and preincubation lengths (0.5 to 22 hours), so as to enhance sperm parameters and achieve capacitation prior to assay. This enabled the examination of different approaches that could contribute in assay improvement but rendered great interlaboratory inconsistency and ambiguous results over efficiency. In addition, the majority of studies did not address neither female factor and potential oocyte deficiency that could affect IVF outcome, nor the period of abstinence of the participants.

Analysis of data indicated 7 studies to have presented SPA results that correlate well with IVF outcome [23, 28, 32–36], indicating this assay to be a valuable prognostic tool when evaluating sperm function. The drawback is that these studies fluctuate extensively in specificity (44–100%) with wide variation in preparation methods, capacitation medium, preincubation lengths, variation in SPA cutoff values (10–20%), and fertilization rate cutoff values. Others have suggested a relative predictive value of SPA at some level, but data analysis failed to present the anticipated values to establish this assay as a valuable predictor of fertilization outcome [24, 26, 29]. Conversely, the clinical relevance of SPA has been debated, and significant false-positive/negative rates were demonstrated [25, 27, 31], suggesting that this assay is of limited predictive value and unreliable for IVF. Furthermore, Kruger et al. [30] stated that, although a negative SPA might indicate male factor infertility, this assay could not be justified as a better predictor than sperm morphology assessment in IVF outcome. Other studies that investigated efficacy on a different setting than the one examined on this review could not validate SPA as a better predictor than computer assisted semen analysis (CASA), manual motility scoring, or even conventional semen analysis [47, 48]. 

The predictive power of HZA was reviewed along with possible drawbacks of this technique and protocol standardization. As summarized by Yao et al. [49], some degree of variation in HZA outcome could be induced by oocyte source and preservation method, sperm concentration and preparation method, base medium and aspiration pipette diameter, and oocyte microdissection method. Consequently, some modifications of the initial protocol were proposed [16, 17, 50–52]. Initial testing with a range of HZA cutoff values (15–36%) did not enable the standardization of a baseline value; however, some range reduction was later achieved. Patient characteristics are summarized in Table 1(b), in the form of normal/abnormal sperm parameters, and the variable oocyte sources exploited by different studies are demonstrated. There are variable but limited sources of zonae, including ovarian tissue from surgical cases or cadavers and noninseminated/unfertilized oocytes donated by IVF patients. In an attempt to provide an alternative oocyte source for the assay, species like gibbons and gorillas were investigated in terms of the oocyte ability to interact with human spermatozoa [53–55], but this process is highly species specific, and none of these options was further explored. Subsequently, a major drawback is the limited availability of human oocytes, a problem which could be circumvented with the possible availability of biologically active recombinant human zona as a substitute, along with other chemical/biological candidates [56–59]. 

The predictive value of HZA has been extensively investigated in terms of IVF outcome and the sum of the studies included in this review served to validate this assay as a predictor of fertilizing capacity [37–43]. A variable range of HZI threshold values (23–36%) was noted (most studies employed either 30% or 36%), and there was some variation in fertilization rate cutoff values (50–66%) and in oocyte source, but the analysis of the published data of the selected studies for this review consistently reported high sensitivity and specificity, with concomitantly high PPV and NPV (Table 3). Increased diagnostic accuracy values demonstrated throughout indicate that in an IVF setting HZA results are specific to fertility potential, establishing whether spermatozoa of the male under investigation are functionally competent to interact with the oocyte and complete a sequence of actions essential for fertilization. 

## 4. Discussion 

Analysis of the published evidence on the preselected sperm function tests validity demonstrated inconsistent results on SPA's role in male fertility evaluation, while HZA was consistently efficient in predicting IVF outcome. When reviewing data on SPA, considerable variation was noted in diagnostic accuracy values with wide sensitivity (52–100%), specificity (0–100%), and PPV (18–100%) and NPV (0–100%) together with fluctuation and notable differentiation in methodology and cutoff values employed by each group. On the other hand, HZA methodology was overall consistent with minor variation in cutoff values and oocyte source, while data analysis reported strong correlation of HZA results with IVF outcome. These studies consistently reported high HZA sensitivity (75−100%), good specificity (57–100%), and high PPV (79–100%) and NPV (68–100%).

On the level of association of SPA with IVF outcome [23–36], some studies reported good correlation, and others could not validate any apparent connection, whereas the rest determined a relative predictive value under specific conditions. Unsurprisingly, these contrasting views have brought about uncertainty over the power of this tool in male fertility assessment. The relevant meta-analyses [60, 61] confirmed the heterogeneity among studies and overall low predictive power of SPA on IVF outcome with increased false-positives and, therefore, reduced specificity. In particular, Mol et al. [60] demonstrated significant heterogeneity of the assay with low sensitivity (37%) and, interestingly, high specificity (95%) and concluded that SPA is an inadequate assay for selecting patients for IVF treatment. Similarly, Oehninger et al. [61] reported a high false-positive rate and thus reduced specificity of SPA as a predictor of fertilization, corroborated also by the European Society of Human Reproduction and Embryology [62], which accordingly recommended its abandonment at that point. Compared with much simpler approaches, such as sperm motility assessment, strict morphology, or even conventional seminology, SPA did not appear to be superior [30, 47, 48]. SPA is still a widely applied research tool as it examines certain functional aspects, but it is currently of limited value in clinical practice, since it does not contribute significant information in male fertility status. Standardised protocols may provide diagnostic information with greater precision in terms of sensitivity and specificity, as well as replicating gamete physiological interaction more accurately, and this is in agreement with Oehninger et al. [61] suggesting that SPA methodology should be revisited to produce a consensus protocol. 

HZA has undergone little variation since its introduction and consistency of methodology are preserved to a great extent across IVF laboratories. A considerable limitation in the applicability of the assay is the restricted reserve of human oocytes [42], although the prospective utilization of biologically active recombinant human zona pellucida could circumvent it. Furthermore, the complexity of performing oocyte dissection into two equal hemispheres requires high accuracy to avoid the potential risk of differentially exposed area between control and test samples. Finally, ethical concerns arise over the extent of the allowed gamete interaction during sperm function assays, although HZA-in contrast to the CZB-exploits oocytes devoid of ooplasm and its application could nullify any points of consideration.

In our review, HZA appeared to be an important marker of spermatozoa fertilizing capacity. It correlated well with IVF outcome, as it demonstrated better sensitivity/specificity and positive/negative predictive power than SPA, in agreement with the findings of a previous meta-analysis [61], reporting high PPV and NPV and consistently low false-negative rates. The noted result of homogeneity of the included studies, together with the subtle methodology variation between laboratories, indicates that HZA is a reliable candidate to assess sperm function capacity. Males with normal or slightly abnormal semen profiles could benefit by this intervention and be evaluated prior to referral to assisted reproduction. This assay provides essential information on several aspects of sperm function and could be used in a sequential fashion with semen analysis and potentially other bioassays in an ART setting. 

The limitations of the current review reflect the corresponding limitations of the individual studies. Although numerous records were originally retrieved during our search, studies that used different assisted conception methods such as intrauterine insemination (IUI) or ICSI, or with primary outcomes such as live birth rate, or pregnancy rate were excluded to preserve consistency over the examined parameters defining the outcome of each study. This was also considered in the context of the exact process that reflects functional capacity of the spermatozoon where fertilization is the outmost measure compared to pregnancy and live birth where other factors such as chromosomal integrity of the spermatozoon, embryo abnormalities, and pregnancy complications could potentially have a major impact on these outcomes. In the same context, protocols including pretreatments or interventions that could exogenously affect sperm function were excluded, in an attempt to signify the value of these tests in a clear-cut manner. Most of the included studies were published over two decades ago—when these techniques were considered breakthrough and were extensively investigated—except that at this period publication criteria differed significantly, leading to missing crucial information and statistical reporting methods relying on the authors' discretion. This limited our ability to explore the different approaches of the assay protocols, the efficiency of the interventions used and participant characteristics to assist direct comparison and possible attempts to communicate with the authors for further clarification were abandoned due to the restricted contact information published at that point. Moreover, this generated the necessity to recalculate diagnostic accuracy values wherever the data sets were available and led to the exclusion of studies that did not report the appropriate statistical values or corresponding data sets. A possible limitation could be attributed with regard to cost efficiency of the particular sperm function tests in practise, but this parameter was not included in our initial goals towards investigation as the core purpose of this review was to clarify the efficiency of these tests in terms of IVF outcome. A previously published review and meta-analysis [61] explored various sperm function tests in a similar manner, although this group did not include the sum of the studies reported here and no direct comparison was performed between these two tests to clarify their definite validity on male fertility investigation. Unavoidably, a number of studies coincide with Oehninger et al. [61] however, calculation of diagnostic accuracy values revealed some degree of discordance in terms of the reported specificity, sensitivity, and PPV and NPV. 

Current clinical opinion relies mostly on conventional semen parameters upon deciding wether to allow natural conception or to refer for IVF treatment and, quite frequently, without identifying male fertility potential or any underlying pathology. Semen analysis is sometimes subject to the evaluators' individual judgement and has been implicated to exert limited predictive value in determining sperm function and fertility potential, as demonstrated by the noted discrepancies in cases when men with normal semen parameters have repeated fertilization failure, while men with very abnormal semen characteristics have fathered children. Some fertility clinics incorporate sperm function tests in their advanced investigations, although their clinical significance has been undermined by the introduction of intracytoplasmic sperm injection (ICSI), a technique that effectively circumvents the need for functioning sperm. However, consideration should be given to ethical and biological aspects; should natural selection be allowed to take place avoiding the invasive micromanipulation techniques that mostly rely on morphology in deciding which spermatozoon is the most fit to fertilize an oocyte. 

Impaired spermatozoa interaction with the zona pellucida and oocyte investments has been classified amongst the principal causes of fertilization failure, and, in this context, our data analysis highlighted the validity and high predictive value of HZA; this result situates this assay within the criteria for plausibility and applicability of diagnostic tests. From a clinical perspective, this technically demanding technique requires an affirmed oocyte source, or the commercial availability of biologically active human recombinant zona pellucida, to confront limited oocyte availability. In contrast, SPA failed to produce uniform results on IVF outcome prediction, and this finding along with protocol inconsistency, indicate, that it is far from being an absolute test for male fertility potential. However, more effort should be devoted in optimizing and reevaluating this technique as a great advantage of SPA is that this assay utilizes hamster oocytes are more readily available than human and clinical application of SPA could possibly be more realistic than implementing an assay that requires resources which are currently limited.

Ideally, sperm function tests should incorporate a wide array of functions in a single assay; however, evidence published to date indicates an apparent dominance of HZA in the context of clinical significance. Following technical optimization and establishment of a reliable oocyte source or analogue availability, HZA could be applied in clinical practice and could be incorporated as part of a range of tests for the profiling of gamete functionality. Future technological advances could promote further improvement of sperm function tests or even the introduction of a single, conclusive test on multiple functional aspects for rapid and accurate diagnosis and optimal clinical management.

## Supplementary Material

Supplemental Figure 1: Overview of the conventional SPA methodology. Hamster ova with chemically removed zona to allow interspecies interaction, are incubated in control and sample droplets with fixed sperm concentration and are microscopically assessed to determine sperm penetration rate.
Supplemental Figure 2: Overview of the conventional HZA methodology. Bisected human oocytes are differentially incubated in control and sample droplets with fixed sperm concentration and are microscopically assessed to determine the Hemizona Index.

## Figures and Tables

**Table:** (a)

Study group	Publication date	Study design	*n*	Normal SP (*n*)	Abnormal SP (*n*)	Capacitation medium	Pre-incubation period
Margalioth et al. [23]	1983	Prospective	20	20	0	NR	NR
Wolf et al. [24]	1983	Prospective	24	24	0	Ham's F-10 with 7,5% maternal serum	3 hrs
Foreman et al. [25]	1984	Prospective	22	14	8	Earle's with 8% HSA	3 hrs
Ausmanas et al. [26]	1985	Prospective	54	42	12	BWW with 35 mg/mL HSA	5 hrs
Belkien et al. [27]	1985	Prospective	29	23	6	Ham's F-10 with 10% maternal serum	6 hrs
Margalioth et al. [28]	1986	Prospective	134	124	10	mBWW with 35 mg/mL HSA	18 hrs
Corson et al. [29]	1987	Prospective	30	17	13	BWW with 5 mg/mL HSA	18–20 hrs
Kruger et al. [30]	1988	Retrospective	84	NR	NR	NR	NR
Coetzee et al. [31]	1989	Prospective	71	NR	NR	BWW	18–20 hrs
Ibrahim et al. [32]	1989	Prospective	59	35	24	BWW with 3.5 mg/mL HSA	3 hrs
Nahhas and Blumenfeld [33]	1989	Prospective	31	27	4	BWW with 36 mg/mL HSA	18 hrs
McClure et al. [34]	1990	Prospective	19	10	9	BWW with 6 mg/mL HSA	18 hrs
						0.4 mL of Human Follicular Fluid	0.5 hrs
Soffer et al. [35]	1992	Follow-up	241	NR	NR	TEST yolk buffer	18–22 hrs
Freeman et al. [36]	2001	Prospective	216	NR	NR	BWW with TEST yolk buffer	18–20 hrs

Abbreviations: *n* (number of participants/patients), SP (Semen Parameters), BWW (Biggers, Whitten and Whittingham medium), HSA (Human Serum Albumin), NR (Not Reported).

**Table:** (b)

Study group	Publication date	Study design	*n*	Normal SP (*n*)	Abnormal SP (*n*)	Oocyte source	Oocyte preservation
Oehninger et al. [37]	1989	Prospective	28	17	11	SR ovarian tissue	Salt storage
Oehninger et al. [38]	1991	Prospective	37	15	22	IVF patient donation	Salt storage
Oehninger et al. [39]	1992	Prospective	44	31	13	SR ovarian tissue	Salt storage
Franken et al. [40]	1993	Prospective	112	58	54	SR ovarian tissue and IVF patient donation	Salt storage
Franken et al. [41]	1993	Prospective	48	28	20	SR ovarian tissue and IVF patient donation	Salt Storage
Gamzu et al. [42]	1994	Prospective	65	47	18	IVF patient donation	Salt Storage
Oehninger et al. [43]	1997	Prospective	196	138	58	SR ovarian tissue	Salt storage

Abbreviations: *n* (number of participants/patients), SP (Semen Parameters), SR (Surgically Recovered).

**Table 2:** SPA and IVF outcome and summary of diagnostic accuracy findings.

Study group, date	SPA threshold (%)	Fertilization rate cut-off (%)	Sensitivity	Specificity	PPV	NPV
Margalioth et al., 1983 [23]	≥20	>0	100%	70%	77%	100%
Wolf et al., 1983 [24]	≥10	>0	88%	0%	68%	0%
Foreman et al., 1984 [25]	>10	>0	67%	53%	18%	91%
Ausmanas et al., 1985 [26]	>15	>0	73%	33%	95%	7%
Belkien et al., 1985 [27]	>15	>0	87%	17%	80%	25%
Margalioth et al., 1986 [28]	>20	>0	94%	57%	85%	78%
Corson et al., 1987 [29]	≥11	>0	52%	100%	100%	39%
Kruger et al., 1988 [30]	>10	>0	59%	62%	82%	33%
Coetzee et al., 1989 [31]	>10	>0	65%	85%	95%	35%
Ibrahim et al., 1989 [32]	>17	>0	74%	84%	NR	NR
Nahhas and Blumenfeld, 1989 [33]	>20	>0	100%	44%	81%	100%
McClure et al. 1990 [34]	>10	>0	93%	75%	93%	75%
Soffer et al., 1992 [35]	>20	>0	96%	NR	82%	74%
Freeman et al., 2001 [36]	≥20	>50	70%	99%	98%	84%

Abbreviations: PPV (positive predictive value), NPV (negative predictive value), and NR (not reported).

**Table 3:** HZA and IVF outcome and summary of diagnostic accuracy findings.

Study group, date	HZI threshold (%)	Fertilization rate cutoff (%)	Sensitivity	Specificity	PPV	NPV
Oehninger et al., 1989 [37]	>36	>65	83%	95%	83%	95%
Oehninger et al., 1991 [38]	>36	>65	80%	100%	100%	85%
Oehninger et al., 1992 [39]	>35	>65	100%	61%	79%	100%
		>0	90%	57%	82%	73%
Franken et al., 1993 [40]	>30	>50	84%	72%	85%	70%
Franken et al., 1993 [41]	>30	>55	75%	75%	81%	68%
Gamzu et al., 1994 [42]	>23	>66	100%	94%	85%	100%
Oehninger et al., 1997 [43]	>30	>60	93%	73%	85%	87%

Abbreviations: HZI (hemizona index), PPV (positive predictive value), and NPV (negative predictive value).
