# Supplementary material for: Hemizona Assay and Sperm Penetration Assay in the Prediction of IVF Outcome: A Systematic Review
Source: Biomed Res Int. 2013 Oct 21;2013:945825. doi: 10.1155/2013/945825 (PMC3818817; doi:10.1155/2013/945825)
Supplement: Supplementary file 1 — Supplemental Figure 1: Overview of the conventional SPA methodology. Hamster ova with chemically removed zona to allow interspecies interaction, are incubated in control and sample droplets with fixed sperm concentration and are microscopically assessed to determine sperm penetration rate. Supplemental Figure 2: Overview of the conventional HZA methodology. Bisected human oocytes are differentially incubated in control and sample droplets with fixed sperm concentration and are microscopically assessed to determine the Hemizona Index. [file 945825.f1.ppt]

## Slide 1
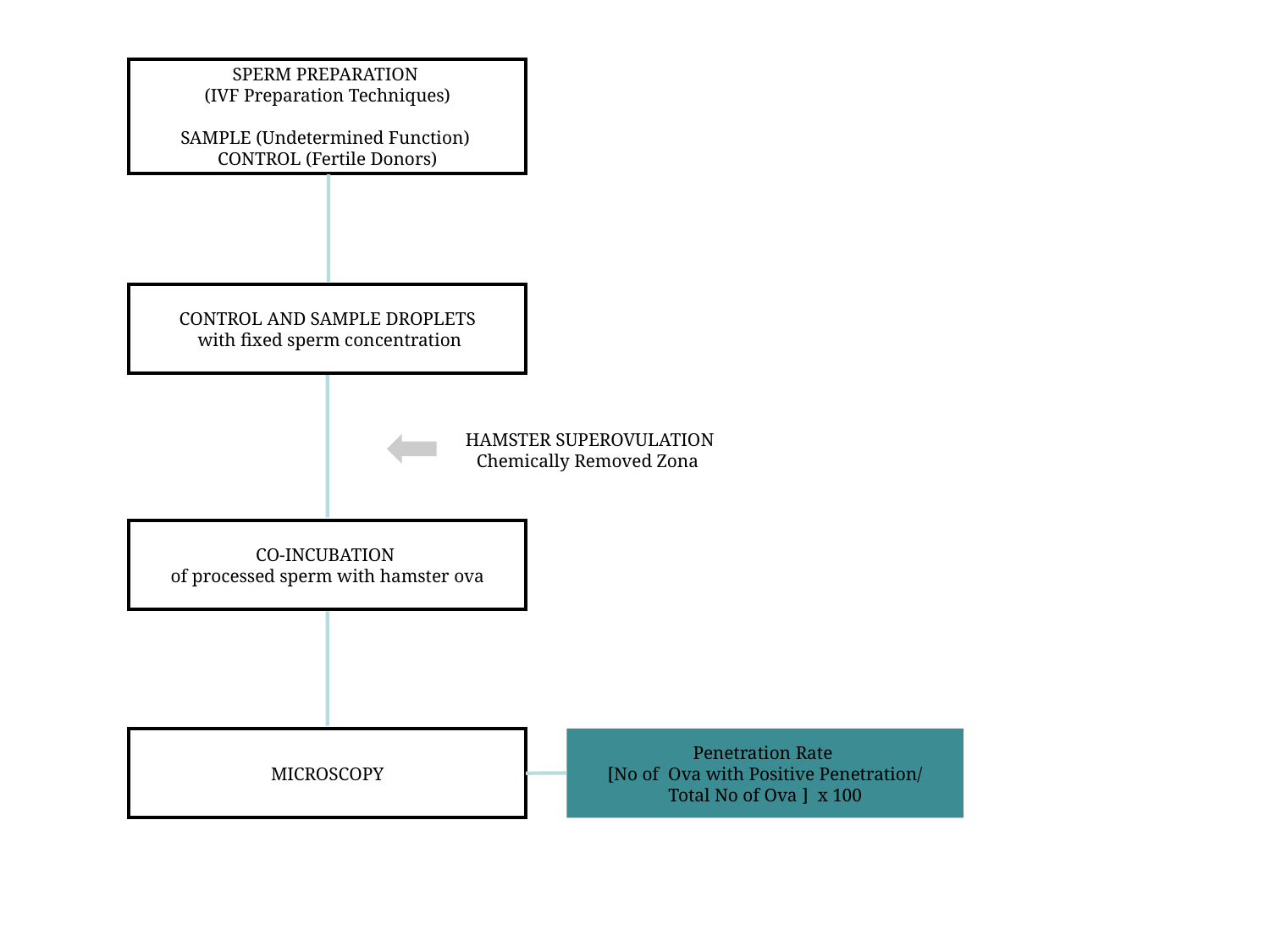

SPERM PREPARATION
(IVF Preparation Techniques)
SAMPLE (Undetermined Function)
CONTROL (Fertile Donors)
CONTROL AND SAMPLE DROPLETS
 with fixed sperm concentration
HAMSTER SUPEROVULATION
Chemically Removed Zona
CO-INCUBATION
of processed sperm with hamster ova
MICROSCOPY
Penetration Rate
 [No of Ova with Positive Penetration/
Total No of Ova ] x 100
