# Supplementary material for: Hemizona Assay and Sperm Penetration Assay in the Prediction of IVF Outcome: A Systematic Review
Source: Biomed Res Int. 2013 Oct 21;2013:945825. doi: 10.1155/2013/945825 (PMC3818817; doi:10.1155/2013/945825)
Supplement: Supplementary file 2 [file 945825.f2.ppt]

## Slide 1
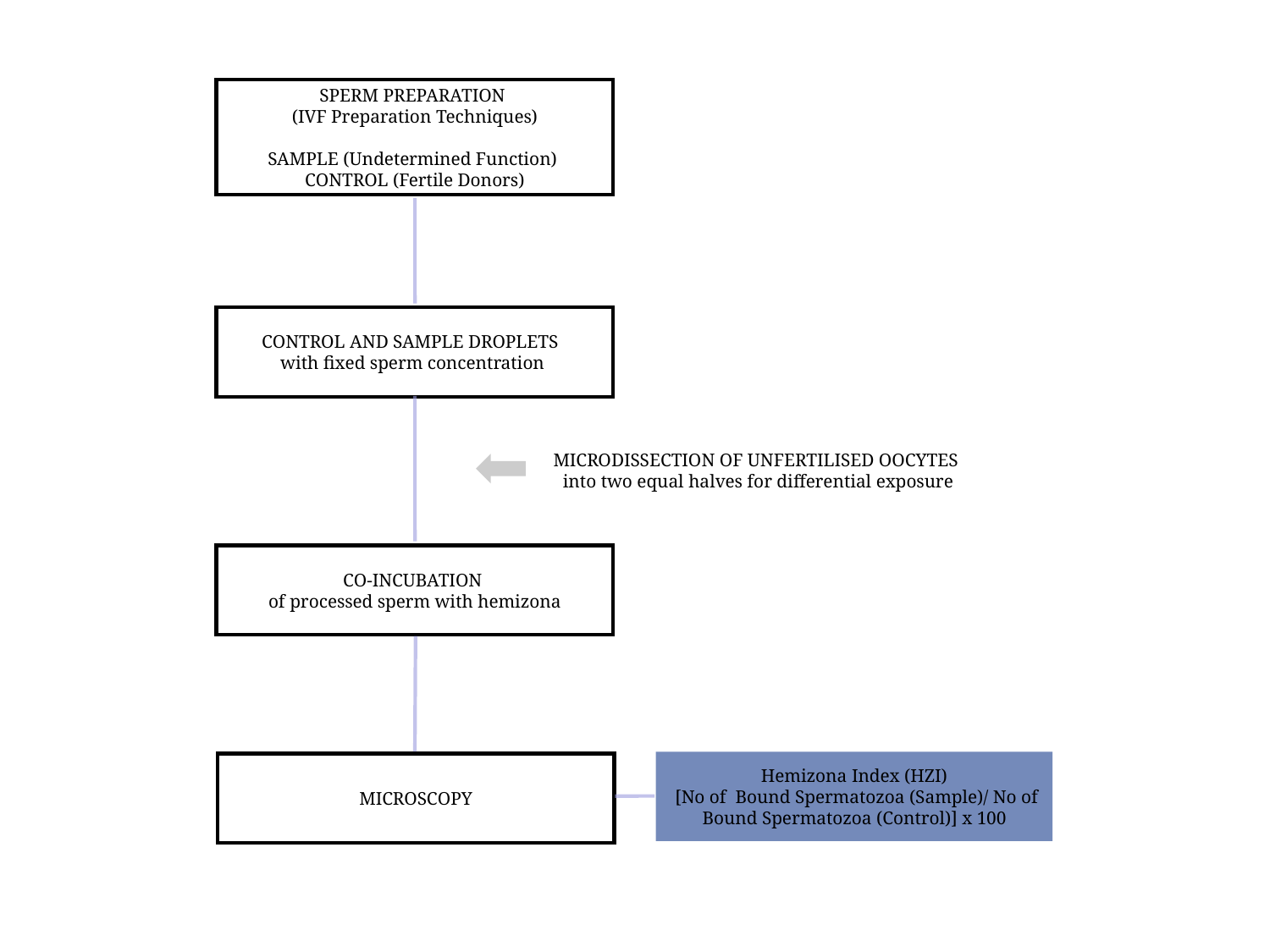

SPERM PREPARATION
(IVF Preparation Techniques)
SAMPLE (Undetermined Function)
CONTROL (Fertile Donors)
CONTROL AND SAMPLE DROPLETS
with fixed sperm concentration
MICRODISSECTION OF UNFERTILISED OOCYTES
into two equal halves for differential exposure
CO-INCUBATION
of processed sperm with hemizona
Hemizona Index (HZI)
 [No of Bound Spermatozoa (Sample)/ No of Bound Spermatozoa (Control)] x 100
MICROSCOPY
